# Mass Spectrometry of Bis-Quinolizidine Alkaloids: FAB-MS of Oxo-Substituted Sparteines

**DOI:** 10.1155/2011/652589

**Published:** 2011-06-05

**Authors:** Beata Jasiewicz, Elżbieta Wyrzykiewicz

**Affiliations:** Faculty of Chemistry, Adam Mickiewicz University, Grunwaldzka 6, 60-780 Poznań, Poland

## Abstract

The unpublished in the literature FAB mass spectral fragmentation of seven oxosparteines (i.e., 2-oxosparteine, 15-oxosparteine, 17-oxosparteine, 2,17-dioxosparteine, 2,13-dioxosparteine, 2-oxo-13-hydroxysparteine, and 2-oxo-17-hydroxysparteine) is investigated. Fragmentation pathways, elucidation of which was assisted by FAB/collision-induced dissociation (CID) mass spectra measurements, are discussed. The data obtained create the basis for distinguishing positional isomers.

## 1. Introduction

Bis-quinolizidine alkaloids produced by Lupine species have generated much interest because of their valuable pharmacological properties. Both pharmacological and toxicological properties of these alkaloids are well known [1, 2]. Sparteine appears to offer protection to plants from *Leguminosae* family against insects and grazing mammals [3, 4]. Several bis-quinolizidine alkaloids (sparteine, lupanine, 17-oxosparteine, 13-hydroxylupanine, angustifoline, etc.) show antihypertensive, antipyretic, anti-inflammatory, antiarrhythmic, diuretic, hypoglicemic, hypotensive, antidiabetic, respiratory depressant and stimulant, and uterotonic properties [5, 6]. The mass spectrometry study of bis-quinolizidine alkaloids has been stimulated by the evidence of the method's ability to distinguish their stereoisomers, metamers, and positional isomers [7–14]. The main characteristic of the so-called “hard” electron-impact induced ionization (EI) of mass fragmentation of bis-quinolizidine alkaloid molecular ions is the dependence of the fragmentation pathway of the bis-quinolizidine skeleton on the stereochemistry of the A/B and C/D ring junctions. The stereochemical effects that are encountered with dissociations of stereoisomers incorporating saturated heterocycles rings are due to the ability of chemical bonds to be broken or formed. Mass spectrometry includes a broad range of techniques that have allowed us to prove the detailed structures of organic compounds in a variety of ways. 

Fast atom bombardment ionization (FAB) is classified as a soft ionization technique in mass spectrometry and is well suited to organic compounds which contain a basic functional group. Those compounds tend to run well in positive ion mode. In the positive FAB technique a high velocity, rare gas atom molecular beam was produced in the ionization source, and directed onto the sample which was in solution (in the matrix) on a target, thus causing desorption of protonated molecular ions from the sample. Generally, positive FAB produces protonated molecular ions M+H^*⌉*+^ with a little fragmentation, and so the spectra may be expected to be readily interpretable. There are some limitations because the presence of matrix gives rise to matrix-related ions. The matrix produces a characteristic spectrum which will quickly become familiar. If, by chance, the sample give rise to ions at anyone of the *m/z* values of matrix, than the matrix should be changed. There are many references in the literature for different matrices where their molecular formulae and masses, their most frequently encountered *m/z* ions, and their uses have been summarized. 

As a continuation of our previous study [15] it seems reasonable to extend investigation over FAB mass fragmentation of isomeric oxo-substituted sparteine derivatives. FAB-MS gives useful information for the chemical characterization of different types of alkaloids [16, 17]. Fast atom bombardment (FAB) mass spectral behaviour of oxo-substituted sparteine derivatives has not been reported in the literature. In a previous work [15] we described the fragmentation routes of seven bis-quinolizidine alkaloids under fast atom bombardment conditions. We have shown that positional isomers of sparteine derivatives can be differentiated on the basis of their FAB mass spectra. 

**Figure 1:**
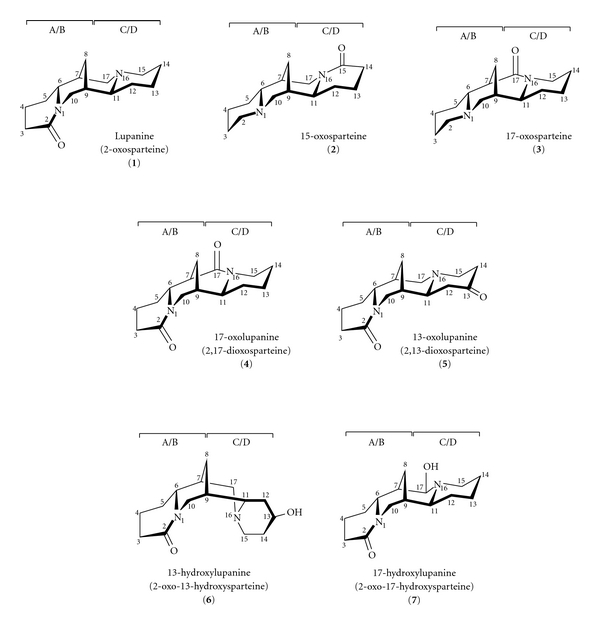
Structures of sparteine lactams (**1**–**3**), oxo-substituted derivatives of lupanine (**4**, **5**) and hydroxy-substituted derivatives of lupanine (**6**, **7**).

The aim of this study was to explain the FAB mass fragmentation of isomeric sparteine lactams, for example, 2-oxosparteine (lupanine) **1**, 15-oxosparteine **2**, 17-oxosparteine **3**, 2,17-dioxosparteine (17-oxolupanine) **4**, 2,13-dioxosparteine (13-oxolupanine) **5**, 2-oxo-13-hydroxysparteine (13-hydroxylupanine) **6**, and 2-oxo-17-hydroxysparteine (17-hydroxylupanine) **7** (Figure 1). These compounds consist of four rings, two of which (A/B) form a sofa/chair system of *trans* quinolizidine, the second *trans* system (C/D) form a boat/chair conformations. On the basis of the analysis of the fragmentation processes of **1**–**7** in the FAB conditions we wished to establish whether it would be possible to distinguish the positional isomers (**1**–**3**; **4**,**5**; **6**,**7**) (Figure 1). 

## 2. Experimental Section

Compounds **1**–**7 **were obtained in the form of free basis according to literature methods [18–24]. Their spectral characteristics were consistent with the literature data [18–24]. The FAB spectra were produced using 3-nitrobenzyl alcohol (m-NBA) as matrix. These spectra were recorded in positive mode on an AMD-Intectra GmbH Harpstedt D-27243 model 604 two-sector mass spectrometer. For the collision-induced dissociation (CID) experiments, helium was used as a collision gas in the first field-free region (1FFR) at a pressure corresponding to 50% attenuation of the precursor ion signal.

## 3. Results and Discussion

The mass spectrometric behaviour of isomeric **1**–**7** was investigated in details by the positive FAB mass spectrometry combined with CID. The relative abundances of characteristic peaks of even-electron as well as matrix-derived ions are presented in Table 1. 

**Scheme 1:**
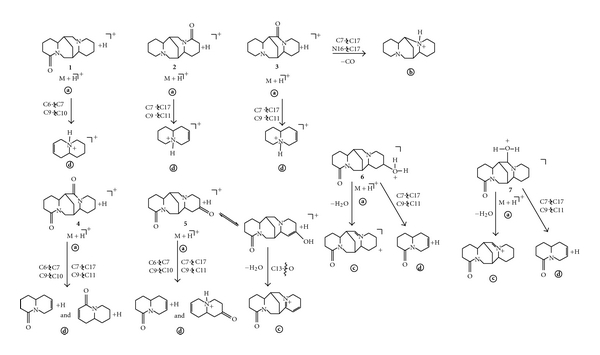
The fragmentation pathways in the FAB spectra of **1**–**7**.

On the basis of FAB and FAB/CID mass spectra of **1**–**7**, the FAB mass fragmentations of these compounds are shown in Scheme 1. In the FAB spectra of **1**–**7** apart from the expected protonated [M+H]^+•^ **a **ions, there are also fragment ions. Fragmentation of the cyclic M+H^*⌉*+^ **a** of **1**, **2**, and **3** (Scheme 1, Table 1) proceeds by the cleavages of two bonds of ring B and C of the sparteine skeleton. The cleavages of C6-C7 and C9-C10 bonds of ring B (for **1**) or C9-C11 and C7-C17 ones of ring D (for **2** and **3**) lead to the even-electron fragment ions **d** [C_9_H_15_N+H]^+^, at *m/z* 138. The cleavages of C7-C17 and N16-C17 bonds of ring C (for **3**) lead to the even-electron fragment ion **b** [C_14_H_24_N_2_+H]^+^ [M+H-CO]^+^, at *m/z* 221. It should be pointed out that the origin of the even-electron fragment ions **b** and **d** has been confirmed by the FAB/CID mass spectra of **1**–**3**. The even-electron fragment ions **a** [M+H]^+^ gives the base peaks for the spectra of **2** and **3**. In the FAB mass spectrum of **1** the base peak is the even-electron ion NBA+H^*⌉*+^. 

Fragmentation of the cyclic protonated molecule M+H^*⌉*+^ of **3** (Table 1) proceeds also by the cleavages of N16-C17 and C17-C7 bonds of ring C with elimination of the neutral molecule of carbon monoxide and leads to the even-electron fragment ion **b** [C_14_H_24_N_2_+H]^+^ [M+H-CO]^+^ at *m/z* 221.

It ought to be pointed out that in the molecules **2** and **3** in the bis-quinolizidine skeleton A and B rings form a *trans* double-chair system that is relatively resistant (for thermodynamic reasons) to conformational-configurational changes than A/B *trans-*fused rings with sofa/chair conformation of the molecule of **1**. This suggests that the structure of **2** and **3** increases the stability of M+H^*⌉*+^ ions of these compounds in comparison with that of M+H^*⌉*+^ ion of **1**. On the other hand the localization of oxo groups in the different position in bis-quinolizidine skeleton, that is, at C2 (ring A; **1**), at C15 (ring D; **2**) and C17 (ring C; **3**) influences clearly on the elimination of the neutral molecule of carbon monoxide from M+H^*⌉*+^ ion of **3**. Such ejection of CO has been seen previously in the EI mass fragmentation of the molecular ion of 17-oxosparteine [9]. 

The differences in the relative abundances of ions **a** in the spectra of **1**–**3** depend clearly on the differences in the conformations of bis-quinolizidine skeleton of these compounds. 

In the light of these data isomeric compounds **1**, **2**, and **3** can be distinguished from each other on the basis of the differences in the relative abundances of ions **a** and **d** (Table 1, Scheme 1) as well as the presence of the even-electron fragment ion **b** [M+H-CO]^+^ in the FAB mass spectrum of **3.**

The common characteristic features of the FAB fragmentation of the protonated molecules [M+H]^+^ **a** of **4** and **5** are the cleavages of bonds of rings B and C (C6-C7 and C9-C10 as well as C7-C17 and C9-C11, resp.). The fragment ions **d** [C_9_H_12_NO+H]^+•^ at *m/z* 151 were obtained in this way by FAB fragmentation (Table 1, Scheme 1). It should be pointed out that the even-electron ions [M+H]^+^ are the base peaks in the FAB spectra of **4** and **5**. In the FAB fragmentation of **5** (Table 1, Scheme 1) the elimination of a neutral molecule of water from the enol-tautomeric form of the [M+H]^+^ molecule of **5**, that is, the cleavage of C13-O bond of ring D leads to the even-electron fragment ion **c**. Isomers **4** and **5** can be distinguished by the presence of even-electron fragment ion [M+H-H_2_O]^+^ **c** [C_15_H_20_N_2_O+H]^+^ at *m/z* 245 in the FAB spectrum of **5**. 

**Figure 2:**
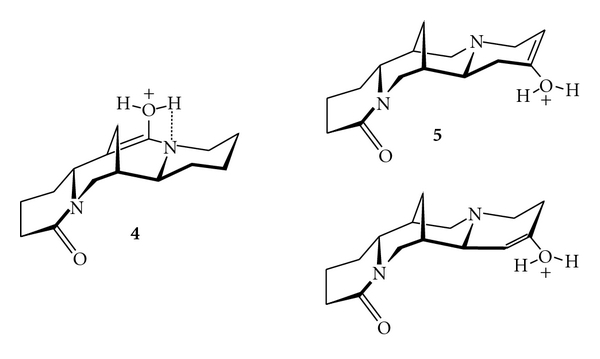
Structures of tautomeric enol forms of **4** and **5**.

The absence of the elimination of water in the FAB mass fragmentation of M+H^*⌉*+^ **a** ion of **4** is probably causes by neighbouring heteroatom participation in the elimination of this small molecule. Protonated **4** shows no fragmentation by loss of water than does protonated **5**. In **4** the proton forms a hydrogen-bridge between oxygen a nitrogen atom of enol-tautomeric form of **4** thus stabilizing the ion. No such stabilization is possible in enol-tautomeric forms of **5** which undergoes fast elimination of water (Figure 2). 

The common characteristic features of the FAB fragmentation of **6** and **7** are the cleavages of C7-C17 and C9-C11 bonds of ring C of the lupanine skeleton. The fragment ions **d** [C_9_H_12_NO+H]^+•^ at *m/z* 151 are obtained in this way of FAB fragmentation. In the FAB mass spectra of **6** and **7** there are also even-electron fragment ions **c** [C_15_H_22_N_2_O][M+H-H_2_O] at *m/z* 247. In the FAB mass spectrum of **6** the base peak is ion [M+H]^+^ **a** and in the FAB mass spectrum of **7** the base peak is ion **c**. The differences in the relative abundances of ions **a**, **c**, and **d** in the FAB spectra **6** and **7** allow differentiation of these positional isomers. 

It ought to be pointed out that the water loss is much more favourable in the FAB mass fragmentation of **7** than **6** because after this elimination in the even-electron fragment ion **c** of **7** the charge is probably situated on the annular nitrogen atom N16, and in the case of **6** the charge is probably situated on the carbon atom C13. 

## 4. Conclusions

Identification and structural characterisation of isomeric bis-quinolizidine alkaloids is an important problem in their analysis. Mass spectrometry is a powerful tool for unambiguous determination of the structure of these compounds. In the literature that is no information about the FAB mass fragmentation of bis-quinolizidine alkaloids. 

The present study has demonstrated that FAB mass fragmentation of isomers of lactams of sparteine sparteine **1**–**7 **(**1**–**3**; **4**, **5**; **6**, **7**) could be expressed as follows. 

(1) The FAB mass fragmentation of the protonated molecules M+H^*⌉*+^ of **1**–**7** proceeds mainly by the cleavages of bonds in B (C6-C7 and N1-C10) ring and C (C9-C11, C7-C17) ring of the bis-quinolizidine skeleton (Table 1, Scheme 1).

(2) The protonated molecules M+H^*⌉*+^ of investigated isomers **1**–**7** follow the common fragmentation pathways, but with differences in the relative abundances of fragment ions (Table 1). 

(3) The differences in the relative abundances of fragment ions depend mainly on the location of the carbonyl function in the bis-quinolizidine skeleton.

(4) The differences in the relative abundances of fragmentation ions depend also on the stereochemistry of A/B and C/D ring junctions of investigated **1**–**7**.

(5) The main FAB fragmentation of **3** involves also elimination of the neutral molecule of carbon monoxide [(M+H-CO)]^+^ *m/z* 221 (Table 1). 

 (6) The main FAB mass fragmentation of **6** and **7** involves also elimination of the H_2_O neutral molecule, yielding ions at *m/z* 245 [M+H-H_2_O]^+^ (**5**) and *m/z* 247 [M+H-H_2_O]^+^ (**6**, **7**), respectively (Table 1).

(7) The differences in relative abundances of even-electron ions **a** and **d** (Table 1) in the FAB mass spectra of **1**, **2**, and **3** and the presence of even-electron ion **b** [M+H-CO] in the FAB mass spectrum of **3** allow differentiation of positional isomers **1**, **2**, and **3**.

(8) The presence of even-electron fragment ion [M+H-OH] **c** allow differentiation of positional isomers **4** and **5** (Table 1).

(9) The differences in the relative abundances of ions **a**, **c**, and **d** in the FAB mass spectra of **6** and **7** allow differentiation of these positional isomers. 

## Figures and Tables

**Table 1:** Relative abundances of characteristic ions in the FAB mass spectra of **1**– **7**.

		Elemental	% Relative abundance						
Ion	m/z	composition	**1**	**2**	**3**	**4**	**5**	**6**	**7**
M+H^*⌉ ***+**^ **a**	249	C_15_H_24_N_2_O+H	50	100	100	—	—	—	—
	263	C_15_H_22_N_2_O_2_+H	—	—	—	100	100	—	—
	265	C_15_H_24_N_2_O_2_+H	—	—	—	—	—	100	25
M+H-CO^*⌉ ***+**^ **b**	221	C_14_H_24_N_2_+H	—	—	8	—	—	—	—
M+H- H_2_O^*⌉ ***+**^ **c**	245	C_15_H_20_N_2_O+H	—	—	—	—	10	—	—
	247	C_15_H_22_N_2_O+H						8	100
**d**	138	C_9_H_15_N+H	57	28	7	—	—	—	—
	151	C_9_H_12_NO+H	—	—	—	10	6	3	8
**e**	154	NBA+H	100	46	9	84	55	58	12

**f**	136	[NBA+H]-H_2_O	62	35	15	54	34	39	18

NBA: C_7_H_7_O_3_N.
